# Association between Participation in Outpatient Cardiac Rehabilitation and Self-Reported Receipt of Lifestyle Advice from a Healthcare Provider: Results of a Population-Based Cross-Sectional Survey

**DOI:** 10.1155/2010/541741

**Published:** 2010-12-16

**Authors:** Natalie A. Johnson, Kerry J. Inder, Ben D. Ewald, Erica L. James, Steven J. Bowe

**Affiliations:** ^1^School of Medicine and Public Health, Faculty of Health, The University of Newcastle, Callaghan, Newcastle, NSW 2308, Australia; ^2^Centre for Health Research & Psycho-oncology (CHeRP), The Cancer Council NSW, and Hunter Medical Research Institute, The University of Newcastle, Callaghan, Newcastle, NSW 2308, Australia

## Abstract

We test the hypothesis that the odds of self-reported receipt of lifestyle advice from a health care provider will be lower among outpatient cardiac rehabilitation (OCR) nonattendees and nonreferred patients compared to OCR attendees. Logistic regression was used to analyse cross-sectional data provided by 65% (4971/7678) of patients aged 20 to 84 years discharged from public hospitals with a diagnosis indicating eligibility for OCR between 2002 and 2007. Among respondents, 71% (3518) and 55% (2724) recalled advice regarding physical activity and diet, respectively, while 88% (592/674) of smokers recalled quit advice. OCR attendance was low: 36% (1764) of respondents reported attending OCR, 11% (552) did not attend following referral, and 45% (2217) did not recall being invited. The odds of recalling advice regarding physical activity and diet were significantly lower among OCR nonattendees compared to attendees (OR 0.34, 95% CI 0.21, 0.56 and OR 0.33, 95% CI 0.25, 0.44, resp.) and among nonreferred respondents compared to OCR attendees (OR 0.10, 95% CI 0.07, 0.15 and OR 0.17, 95% CI 0.14, 0.22, resp.). Patients hospitalised for coronary heart disease should be referred to OCR or a suitable alternative to improve recall of lifestyle advice that will reduce the risk of further coronary events.

## 1. Introduction

Coronary heart disease (CHD) is a major cause of death and disability [1–3]. The benefit of lifestyle changes on mortality in patients with CHD has been established [4], and guidelines for the management of acute coronary syndromes recommend that patients be given lifestyle advice that will reduce the risk of further CHD events [5, 6]. 

Health education, counseling, and behaviour modification strategies are core components of inpatient and outpatient cardiac rehabilitation (OCR) programs, which are recommended for all patients with CHD [5–9]. Unfortunately, decreases in the length of stay for cardiac conditions [7, 8] and suboptimal rates of attendance at OCR programs [10–12] have reduced opportunities for the provision of lifestyle advice via these programs. Patients with CHD may also receive lifestyle advice during routine consultations with clinicians after discharge from hospital. However, results of the EUROASPIRE surveys indicate that lifestyle risk factors receive insufficient attention [13]. Barriers to counseling include time limitations and inadequate reimbursement [14, 15]. 

Duration of advice has been shown to be predictive of patient recall of advice in relation to physical activity, diet, and smoking, with an additional minute of discussion being associated with a 2.5-fold increase in recall [16]. As Australian OCR programs generally involve one to three sessions per week over a six- to eight-week period [7, 8], there may be more time available for the provision of lifestyle advice during OCR than during inpatient cardiac rehabilitation programs or routine consultations. This retrospective analysis of data from the Hunter New England Heart and Stroke Register tests the hypothesis that the odds of self-reported receipt of lifestyle advice from a health care provider will be lower among OCR nonattendees and nonreferred patients compared to OCR attendees.

## 2. Materials and Methods

The Hunter Region, located 130 kilometres north of Sydney in New South Wales, Australia, covers 31,000 square kilometres and has a population of almost 650,000 [17]. The Hunter New England Area Health Service divides the Hunter into three clusters (sectors) for management and administration purposes: the metropolitan Greater Newcastle Sector has two tertiary referral hospitals and one district hospital; the semirural Lower Hunter Sector has one rural referral hospital and three district hospitals; the rural Upper Hunter Sector has three district hospitals (http://www.hnehealth.nsw.gov.au/about_us/clusters_and_acute_hospital_networks/clusters).

The Hunter New England Heart and Stroke Register (the Register) used computerized hospital discharge records to identify adults aged 20 to 84 years discharged with qualifying cardiovascular events from hospitals in the Hunter and New England regions. Eligible people received a letter from the Register several months after discharge seeking permission to keep their personal details for data linkage and permission to contact them about future research projects. A questionnaire on risk factors and secondary prevention was also enclosed. 

Data pertaining to Hunter residents discharged from public hospitals in the region between January 2002 and August 2007 with a principal discharge diagnosis (International Classification of Diseases ICD-10-CM codes) of acute myocardial infarction (AMI; I21, I22); unstable angina pectoris (UAP; I20.0); congestive heart failure (CHF; I50), and ischaemic heart disease (IHD; I20, I24, I25) were extracted. Patients without an acute event but undergoing revascularisation (coronary artery bypass graft surgery [CABG] or percutaneous coronary intervention [PCI] including coronary angioplasty and stenting) were included.

Participants completed a postal questionnaire approximately 5 months after discharge from hospital (median: 148 days, 1st quartile: 91 days, 3rd quartile: 271 days). Information regarding advice on lifestyle changes was collected using three questions with the same stem: “Since your admission to hospital have you been advised by a medical person *(e.g., doctor, nurse, physiotherapist, dietician)* to …”: (i) “do any physical activity?” (ii) “follow a modified fat diet?” and (iii) “stop smoking?” Only people who reported smoking in the past 6 months were asked if they had received advice to quit. Response options were “yes” and “no” in each case.

Information regarding OCR was collected using two questions: “Since your hospital admission have you been advised by a medical person *(e.g., doctor, nurse, physiotherapist, dietician)* to attend an outpatient cardiac rehabilitation program?” and “Since your hospital admission have you attended any sessions of an outpatient cardiac rehabilitation program?” The response options were “yes” and “no.” Patients who reported attending any sessions of an OCR program were classified as “attendees,” patients who reported being advised to attend OCR but were not classified as “nonattendees”, and patients who were not advised to attend OCR were classified as “nonreferred”.

The potential confounding variables considered were limited to those collected by the Register. Those obtained from computerized hospital discharge records were gender (male/female), age group (<70/≥70), country of birth (Australia/other), marital status (married or defacto/other), health sector of residence (urban/nonurban), length of stay for the admission preceding survey completion (<4 days/≥4 days), number of events prior to completion of the questionnaire (1/>1), and hospitalization at least once for the following: AMI, UAP, CHF, IHD, and revascularisation (CABG or PCI) (no/yes response to each). 

Information about coronary risk factors and secondary prevention, not available through the computerized hospital discharge records, was obtained by questionnaire. This included a family history of CHD (father, mother, brother, or sister had a diagnosis or died before the age of 70 years of CHD) (no/yes), body mass index ≥30 kg/m^2^ (no/yes), a self-reported history of high blood pressure, diabetes, high cholesterol, atrial fibrillation (no/yes response to each), and whether they had seen a general practitioner or specialist since discharge (no/yes response to both).

The study was approved by the Hunter New England Human Research Ethics Committee (approval number 07/05/16/5.09) and the University of Newcastle Human Research Ethics Committee (approval number H-553-0807). The manager of the Register extracted and coded the data before giving it to the investigators to protect Registrants' privacy. 

The data were analysed using Intercooled Stata versions 10.0 and 11.0 (Stata Corporation, College Station, TX). The characteristics of people who did and did not report receiving advice to (i) “do any physical activity?” (ii) “follow a modified fat diet?” and (iii) “stop smoking” were compared using *χ* ^2^ tests. Multiple logistic regression analyses were used to determine the relationship between participation in OCR and self-reported receipt of advice from a healthcare provider regarding each of the behaviours mentioned above after adjustment for potential confounding variables. Multiple imputation was also performed to examine the impact of missing data. All variables with a *P*-value less than 0.25 in the *χ* ^2^ analyses were included in the logistic models to provide the most complete control of confounding possible within the limits of the data set [18]. All variables were entered simultaneously and retained in the models. The strength of associations was quantified by estimated odds ratios and 95% confidence intervals. Hosmer-Lemeshow goodness-of-fit test results are also reported.

## 3. Results

**Figure 1:**
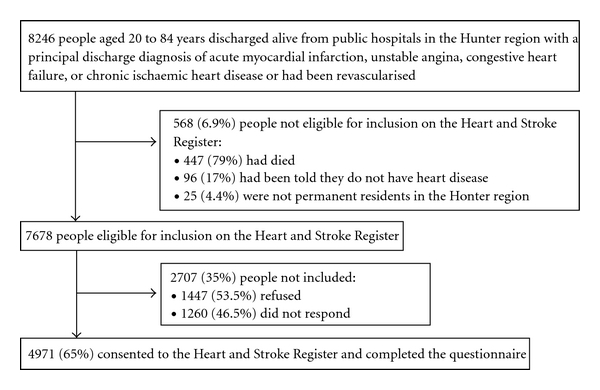
Study Participation Flowchart.

As described elsewhere [10], 8246 people aged 20 to 84 years were discharged from public hospitals in the region with a principal discharge diagnosis of AMI, UAP, CHF or IHD, or following cardiac revascularisation. As shown in Figure 1, 65% (4971/7678) of patients were included in these analyses because they consented to the Register and completed the Heart and Stroke Register questionnaire. Compared with nonconsenters, a greater proportion of consenters were male, married, lived in the urban health sector, and had been revascularised, while smaller proportions had been discharged with the diagnosis of CHF and had only had one event (Table 1).

### 3.1. Self-Reported Receipt of Lifestyle Advice by a Healthcare Provider

Overall, 71% (3518/4971) of respondents reported being advised to participate in physical activity, 24% (1177/4971) did not report receiving this advice, and 5.6% (276/4971) of respondents did not answer this question. Among patients who did and did not report having a physical limitation, 68% (1376/2027) and 81% (1912/2359) reported being advised to participate in physical activity, respectively. Overall, 55% (2724/4971) of respondents reported being advised to follow a modified fat diet, 39% (1921/4971) did not report receiving this advice, and 6.6% (326/4971) of respondents did not answer this question. Almost 14% (674/4971) of respondents reported smoking in the last six months. Of these, 88% (592/674) and 7.6% (51/674) did and did not report being advised to quit, respectively, and 4.6% (31/674) did not answer the question.

### 3.2. Participation in OCR

Among respondents, 36% (1764/4971) reported attending OCR, 11% (552/4971) reported being advised to attend but had not, and 45% (2217/4971) reported they had not been advised to attend OCR. OCR participation status was unknown for 8.8% (438/4971) of respondents who did not answer this question. The proportions who reported receiving lifestyle advice, stratified by OCR participation, are shown in Table 2.

### 3.3. Association between OCR and Self-Reported Receipt of Advice from a Healthcare Provider “to Do Any Physical Activity”

All the variables except country of birth, length of stay, and a self-reported history of high blood pressure had a *P*-value less than  .25 in the *χ* ^2^ analyses and were included in the logistic regression analyses. Missing data ranged from a minimum of 4.0% for Seen GP Since Discharge to a maximum of 13% for Seen Specialist Since Discharge. Among respondents with complete data, 79% (2006/2535) reported being advised to participate in physical activity by a healthcare provider and 21% (529/2535) did not. OCR was significantly associated with self-reported receipt of advice to participate in physical activity after adjustment for potential confounders (Table 3). The Hosmer-Lemeshow goodness-of-fit test indicated that the complete case analysis model fitted the data well (*χ* ^2^ = 6.49, df = 8, *P* = .59). Overall, 76% of the cases were classified correctly.

### 3.4. Association between OCR and Self-Reported Receipt of Advice from a Healthcare Provider “to Follow a Modified Fat Diet”

All the variables except country of birth and length of stay were included in the logistic regression analysis as they had a *P*-value less than  .25 in the *χ* ^2^ analyses. Missing data ranged from a minimum of 2.5% for Told High Blood Pressure to a maximum of 12% for Seen Specialist Since Discharge. Among respondents with complete data, 63% (1691/2676) reported being advised to follow a modified fat diet by healthcare provider and 37% (985/2676) did not. OCR was significantly associated with self-reported receipt of advice to follow a modified fat diet after adjustment for potential confounders (Table 4). The Hosmer-Lemeshow goodness-of-fit test indicated that the complete case analysis model fitted the data well (*χ* ^2^ = 3.59, df = 8, *P* = .89). Overall, 73% of the cases were classified correctly.

### 3.5. Association between OCR and Self-Reported Receipt of Advice from a Healthcare Provider “to Stop Smoking”

Sex, age group, health sector of residence, length of stay for the admission preceding survey completion, at least one admission for AMI, at least one admission for CHF, family history of CHD, body mass index, self-reported history of high blood pressure, self-reported history of atrial fibrillation, and having seen a general practitioner since discharge had a *P*-value less than  .25 in the *χ* ^2^ analyses and were included in the logistic regression analyses. Missing data ranged from a minimum of 2.3% for Told High Blood Pressure to a maximum of 9.3% for Told Atrial Fibrillation. Among respondents with complete data, 92% (446/483) reported being advised to stop smoking by a healthcare provider and 8.1% (37/446) did not. OCR was significantly associated with self-reported receipt of advice to stop smoking after adjustment for potential confounders (Table 5). The Hosmer-Lemeshow goodness-of-fit test indicated that the complete case analysis model fitted the data well (*χ* ^2^ = 3.45, df = 8, *P* = .90). Overall, 76% of the cases were classified correctly.

## 4. Discussion

This retrospective analysis of data collected by the Hunter New England Heart and Stroke Register showed that 55% of patients hospitalised for CHD reported advice from a healthcare provider to follow a modified fat diet, and 71% reported advice to participate in physical activity. Among patients who reported smoking in the past six months, 88% reported being advised to stop smoking. Thus, while most patients received advice about smoking cessation and physical activity, only slightly more than half reported receiving advice about diet.

Our findings are consistent with the results of a large European study of recall of lifestyle advice among patients with heart failure [19]. In both studies, more than half but fewer than three quarters of the patients reported receiving advice regarding physical activity and dietary fat intake. However, the proportions who recalled advice regarding smoking were different; in our study, 88% of patients recalled this advice compared with 42% of patients with heart failure. As only patients who reported smoking in the past six months were asked about advice to stop smoking in our study, this disparity may be due to a difference in study methodology.

As hypothesised, the odds of recalling advice from a healthcare provider regarding physical activity and diet were lower among the nonattendees and nonreferred patients compared to OCR attendees. The odds of reporting advice from a healthcare provider to be physically active were 67% lower among nonattendees compared to attendees, and 90% lower among nonreferred patients compared to attendees. Similarly, the odds of reporting advice from a healthcare provider to follow a modified fat diet were 67% lower among nonattendees compared with attendees, and 83% lower among nonreferred patients compared to attendees. The odds of recalling lifestyle advice from a healthcare provider regarding smoking were lower, but not significantly, among nonreferred patients compared to OCR attendees.

Only 47% of the respondents recalled being referred to OCR, which is consistent with previous research in the region [20]. Since referral is generally a prerequisite for attendance, we recommend that patients who have not attended OCR as a consequence of nonreferral be identified and advised to attend by their general practitioner. Unfortunately, increasing rates of referral to OCR may increase rates of nonattendance. For example, the American Heart Association's Get With the Guidelines Program-based clinical pathway on referral and enrolment into cardiac rehabilitation after AMI led to a significantly higher referral rate; however, most (66%) of the referred patients did not enrol [21]. In addition, research conducted in the Hunter region has shown that almost 60% of nonreferred patients did not feel they would have benefited from attending OCR which suggests they may not have attended if invited [22]. As a consequence, we recommend that referred patients who do not attend OCR be identified by their general practitioner and encouraged to participate in a home-based cardiac rehabilitation program (e.g., The COACH Program [23]) or referred to allied health services (e.g., a dietitian and an exercise physiologist) as part of a structured process. The latter is a feasible and sustainable change in practice because Medicare Australia provides funding for general practitioners to prepare, and for allied health providers to implement, chronic disease management plans for patients with chronic and complex care needs [24]. To facilitate these changes in practice, we will be investigating the possibility of using the register of heart and stroke admissions to alert general practitioners of patients who require referral to OCR or a suitable alternative. 

Strengths of this study include the large sample and the use of multivariate statistical methodology; however, there are a number of limitations. First, only 65% of eligible patients completed the questionnaire. If the respondents are more health oriented, our study will have overestimated the self-reported receipt of lifestyle advice from a health care provider among patients with CHD. Second, while only patients who reported smoking in the past six months were asked about advice to stop smoking, the appropriateness of nonprovision of advice regarding physical activity and diet could not be established. If, for example, patients who did not recall being advised to “do any physical activity” were physically active already, then not being given this advice would have been appropriate for that person, and our data will have underestimated the proportion of patients recalling advice consistent with current guidelines. Similarly, data on patients advised not to participate in physical activity due to disease severity and were waiting for urgent revascularisation, for example, was not available. Third, the validity and reliability of the Heart and Stroke Register questionnaire is not known. Research verifying patient report of receipt of lifestyle advice from a family physician by direct observation showed that patients recalled less than 50% of discussions about diet, smoking, and exercise [25]. Thus, while we are likely to have underestimated the amount of advice provided, we agree that advice that patients do not recall receiving is unlikely to be beneficial [25]. While it is plausible that patients not interested in attending OCR may be less likely to recall being referred, researchers have recently verified self-report of referral to OCR in 82% of cases [26]. Fourth, there was a considerable amount of missing data. Since analyses based only on complete cases or where the variables with missing data have been excluded have been shown to lead to misleading results, we used multiple imputation to examine the impact of the missing data [27]. As the results were similar, we believe the results of the complete case analysis we have reported are not misleading. Fifth, the direction of significant associations could not be ascertained. Therefore, results of this cross-sectional study could be interpreted to suggest that patients who attend OCR are more likely to recall advice about lifestyle or that people who recall advice about lifestyle are more likely to attend OCR. Last, we did not assess the impact of recall of advice on behaviour.

## 5. Conclusions

Although many respondents reported being advised to make lifestyle changes that will reduce the risk of further coronary events, the proportion of patients receiving or recalling advice in relation to diet, and to a lesser extent physical activity, was suboptimal. As hypothesised, the odds of self-reported receipt of advice from a health care provider regarding diet and physical activity were lower among patients who did not attend OCR or were not referred compared with those who attended. OCR nonattendance and nonreferral were not associated with lower smoking cessation advice rates. Although conclusions about causation are not possible, results of this study suggest that patients who do not attend OCR may miss out on or fail to recall advice on lifestyle changes that will reduce the risk of further CHD events. We therefore recommend that patients hospitalised for CHD who have not attended OCR as a consequence of nonreferral be identified by their general practitioner and advised to attend. In addition, we recommend that patients who have not attended OCR despite referral be identified and encouraged to participate in a home-based cardiac rehabilitation program or be referred to appropriate allied health care providers via the preparation of a chronic disease management plan.

## Figures and Tables

**Table 1:** Demographic and clinical characteristics of consenters and nonconsenters.

Characteristic	Included (*N* = 4971) *N* (%)	Not included (*N* = 2707) *N* (%)	*χ* ^2^; **d** **f** = 1; **P**
Aged 20–69 years	2566 (52)	1393 (51)	0.02; .89
Male	3047 (61)	1484 (55)	30.4; <.001
Born in Australia	3904 (86)	2191 (86)	0.3; .62
Married	2981 (66)	1358 (54)	97.8; <.001
Reside in urban health sector	3477 (70)	1731 (64)	28.9; <.001
One event	3596 (72)	2080 (77)	18.4; <.001
Total length of stay <4 days	2239 (45)	1153 (43)	4.3; .04
Admitted with AMI at least once	1853 (37)	911 (34)	11.7; .003
Admitted with UAP at least once	1934 (39)	870 (32)	34.6; <.001
Admitted with CHF at least once	770 (15)	622 (23)	66.2; <.001
Admitted with IHD at least once	1574 (32)	718 (27)	22.1; <.001
Admitted with stroke	51 (1.0)	30 (1.1)	0.1; .74
Revascularised	1486 (30)	545 (20)	85.8; <.001

AMI, acute myocardial infarction; UAP, unstable angina pectoris; CHF, congestive heart failure; IHD, ischaemic heart disease.

**Table 2:** Self-reported receipt of lifestyle advice from a healthcare provider stratified by participation in outpatient cardiac rehabilitation.

	Advised to“do any physical activity”			Advised to “follow a modified fat diet” (*n* = 4347)	Advised to “stop smoking” (*n* = 600)
	All respondents (*n* = 4330)	Respondents with no physical limitations^a^ (*n* = 2217)	Respondents with physical limitations^b^ (*n* = 1848)		
OCR group					
–Attendees	96 (1626/1701)	96 (1008/1047)	94 (517/549)	84 (1428/1710)	95 (233/245)
– Nonattendees	87 (458/527)	90 (245/271)	83 (185/233)	64 (338/527)	96 (100/104)
– Nonreferred	57 (1197/2102)	62 (554/899)	53 (571/1076)	39 (824/2110)	88 (221/251)

^a^“No” response to the question: “Do you have any physical problems (e.g., arthritis, back problems, or hemiparesis) which stop you from doing any physical activity?”

^b^“Yes” response to the question: “Do you have any physical problems (e.g., arthritis, back problems, or hemiparesis) which stop you from doing any physical activity?”

**Table 3:** Association between participation in outpatient cardiac rehabilitation and self-reported receipt of advice to participate in physical activity.

	Total *N* (% advised to do any physical activity)	Unadjusted odds ratio (95% confidence interval) (*n* = 4330)	Adjusted odds ratio with no imputation (95% confidence interval) (*n*=2535)^a^	Adjusted odds ratio with imputation (95% confidence interval) (*n*=4971)^b^
OCR group				
– Attendees	1701 (96)	1	1	1
– Nonattendees	527 (87)	0.31 (0.22, 0.43)	0.34 (0.21, 0.56)	0.35 (0.25, 0.49)
– Nonreferred	2102 (57)	0.06 (0.05, 0.08)	0.10 (0.07, 0.15)	0.11 (0.08, 0.15)

^a^Complete case analysis.

^b^Multiple imputation method.

**Table 4:** Association between participation in outpatient cardiac rehabilitation and self-reported receipt of advice to follow a modified fat diet.

	Total *N* (% advised to follow a modified fat diet)	Unadjusted odds ratio (95% confidence interval) (*n* = 4347)	Adjusted odds ratio with no imputation (95% confidence interval) (*n*=2676)^a^	Adjusted odds ratio with imputation (95% confidence interval) (*n*=4971)^a^
OCR group				
Attendees	1710 (84)	1	1	1
Nonattendees	527 (64)	0.35 (0.28, 0.44)	0.33 (0.25, 0.44)	0.36 (0.28, 0.46)
Nonreferred	2110 (39)	0.13 (0.11, 0.15)	0.17 (0.14, 0.22)	0.18 (0.15, 0.22)

^a^Complete case analysis.

^b^Multiple imputation method.

**Table 5:** Association between participation in outpatient cardiac rehabilitation and self-reported receipt of advice to quit smoking.

	Total *N* (% advised to stop smoking)	Unadjusted odds ratio (95% confidence interval) (*n* = 600)	Adjusted odds ratio with no imputation (95% confidence interval) (*n*=483)^a^	Adjusted odds ratio with imputation (95% confidence interval) (*n*=674)^b^
OCR group				
Attendees	245 (95)	1	1	1
Nonattendees	104 (96)	1.29 (0.41, 4.09)	0.81 (0.24, 2.79)	1.02 (0.30, 3.49)
Nonreferred	251 (88)	0.38 (0.19, 0.76)	0.48 (0.19, 1.23)	0.51 (0.22, 1.17)

^a^Complete case analysis.

^b^Multiple imputation method.
